# Case management for older women experiencing homelessness in Australia: a sustaining tenancies model of housing and support

**DOI:** 10.3389/fgwh.2025.1724593

**Published:** 2025-12-10

**Authors:** Juliet Watson, Robyn Martin, Freda Haylett

**Affiliations:** 1 Social Equity Research Centre, School of Global, Urban and Social Studies, RMIT University, Melbourne, VIC, Australia; 2 Social Work and Human Services, School of Global, Urban and Social Studies, RMIT University, Melbourne, VIC, Australia; 3 Violet Vines Marshman Centre for Rural Health, La Trobe University, Bendigo, VIC, Australia

**Keywords:** homelessness, housing, older women, case management, social care and housing, case study, housing precarity

## Abstract

This community case study examines the efficacy of the Women's Housing Support Program (WHSP)_,_ which provides case management to older women experiencing homelessness in Melbourne, Australia. In recent years there has been an increase in the number of older women experiencing homelessness in Australia. Some have experienced long-term, chronic homelessness, but there has also been an escalation in homelessness for women who have previously led conventional lives before a significant event such as relationship breakdown, loss of employment, or health crisis results in poverty that contributes to homelessness. The circumstances of older age, gender, and homelessness mean that some older women require specialised responses to access suitable long-term housing and to receive appropriate support that will stabilise their housing. This case study explores the distinctive social, health, and housing needs of women accessing the WHSP and considers how the sustaining tenancies model of support responds to these needs. Based on a mixed-methods study that included interviews with service users, case managers, senior managers, and an external service provider, as well as program data analysis, the case study indicates that older women benefit from specialised support that focuses on housing for life, health care, emotional support, and digital literacy. Additionally, in order to facilitate successful outcomes, this support needs to be flexible, client-centred, and trauma-informed.

## 1 Introduction

Older women experiencing homelessness face particular housing and wellbeing challenges that can require specialised support. The Australian 2016 Census reported a 31 per cent rise in homelessness among women aged 55 and over since the previous 2011 census (1). Although older women comprise six per cent of the total homeless population, the increase represented the sharpest upsurge in homelessness corresponding to age (1), a prevalence that has subsequently stabilised according to the 2021 Census (2). Women also account for 53 per cent of those aged 55 and above seeking support from specialist homelessness services (3).

In Australia, as in other places, definitions of homelessness vary according to application, which may include, for example, policy development, service provision and research objectives. The cultural definition (4) is commonly adopted by the Australian homelessness sector. Cultural homelessness locates literal meanings (such as ‘roofless’ and ‘rough sleeping’) and categories of inadequate housing (such as temporary shelter, ‘couch surfing’, and rooming houses) within socio-historic contexts and community standards (4). Extending this contextual approach, a consideration of gender is essential in how homelessness is framed; otherwise, the trajectories and varied experiences of women are ignored, inadvertently positioning men's experiences as the norm (5). For example, when compared with Australian men aged over 55, women of the same age are less likely to be rough sleeping and more likely to be staying temporarily with other households, in supported accommodation, or inhabiting severely overcrowded dwellings (2).

When women experience homelessness for the first time in later life, this often follows conventional housing histories (6, 7). Disruption to housing stability for older women, as with most people, is associated with poverty. For older women, housing vulnerability is compounded by relationship breakdown (including family violence), death of a partner, interruption to employment, health crisis, and/or an unexpected increase in rent (8–10). The gender pay gap further compromises housing stability as women commonly have lower retirement funds due to time spent out of the paid workforce to raise children, casual or part-time work, and unpaid family carer roles (6, 8, 11, 12)

In Australia, social housing is delivered to people on low incomes who cannot afford private rental accommodation. Social housing consists of state-owned and managed public housing, state-owned and managed Indigenous housing, community housing and Indigenous community housing managed by not-for profit organisations. Waitlists are long with 169,000 households awaiting public housing allocation and 15,100 households awaiting allocation to state-owned and managed Indigenous housing (13), with data unavailable for community and Indigenous community housing waitlists. Priority access to social housing is offered to people: 1) experiencing homelessness and receiving support; 2) escaping or who have escaped family violence; 3) with a disability or significant support needs; and 4) who need to relocate for health reasons (14). This includes older people who may be prioritised for aged care supported housing (14).

Two thirds of primary tenants (269,000 households) in public housing and state-operated and managed Indigenous housing are aged 50 years or older (13). International research indicates that older people in social housing face multiple vulnerabilities including disability, chronic illness, mental health challenges, loneliness, and food insecurity (15). Moreover, the residualisation of public housing and the related tenancy movement, changes to the tenancy composition, and perceived increase in anti-social behaviour contributes to older people not feeling safe and secure in their homes (16, 17). Older people on low incomes also face significant financial burdens that impact their capacity to meet private rental payments and which account for 50–80 per cent of their income (18), resulting in forced cutbacks on essential items such as food and medication (19, 20). This makes single older women especially vulnerable to increasing rental costs that have not been matched by comparable rises in income support, and which must be borne alone (6, 21).

## 2 Context

The Women's Housing Support Program (WHSP), launched in 2022, provides case management to residents at the Lakehouse, which is short-term transitional accommodation for women aged over 50 located in Melbourne, Australia. Although models of transitional housing differ in terms of duration of stay and support offered, in Australia it is typically a form of subsidised rental accommodation for people experiencing homelessness or at risk of becoming homeless; a place where residents can stabilise their housing and, if needed, develop independent living skills and establish supports before moving to long-term housing. However, due to limited affordable housing options, bottlenecks are common, resulting in people remaining in transitional housing far longer than needed (22–24).

Funded by Homes Victoria (Victorian Government), the WHSP was developed, and is delivered, by YWCA (Young Women's Christian Association) Australia and Y Housing. The YWCA partnered with Housing All Australians to establish the Lakehouse. Formerly an aged-care facility, the Lakehouse was repurposed in 2018 as a rooming house with 38 single bedrooms, including ensuite bathrooms, and as a response to insufficient affordable housing options for older women in Melbourne. Specialist support is delivered by the WHSP to around 48 women per year.

## 3 Methods

This community case study is derived from an evaluation of the WHSP undertaken by researchers from RMIT University from June 2022 to November 2023. An evaluation working group comprising YWCA senior managers oversaw the research design and implementation. A mixed methods approach that included qualitative and quantitative data collection was used to document program processes and to appraise the efficacy of the WHSP. Qualitative data collection involved voluntary semi-structured interviews with nine service users, three case managers, four senior managers, and one external service provider. Service user participants were drawn from current residents of the Lakehouse or who had exited in the previous four weeks. The WHSP case managers were responsible for initial recruitment with follow up from the research team if there was interest from the service users. The service users were required to have had a minimum of six weeks engagement with the WHSP, ensuring enough time to reflect on their experiences in the program. The interviews included questions about circumstances prior to entering the WHSP, time in the program, supports and services received, as well as demographic information. Contact details for the case managers were provided by the evaluation working group, with the researchers following up with recruitment. In the interviews, the case managers were asked about their experiences working in the WHSP including what had been achieved, the appropriateness of supports in place for service users, and the program implementation process. The same recruitment process was used for the interviews with senior managers. These interviews were conducted in two stages to capture the evolution of the WHSP over the period of the evaluation. Three senior managers were interviewed in Stage 1 and two senior managers (which included one follow-up consultation) were interviewed in Stage 2. The interviews focused on the implementation of the WHSP model, program outcomes, trauma-informed and culturally-sensitive practice, and replicability of the model. A focus group for external service providers was initially planned, with a contact list made available to the researchers by the evaluation working group. However, a low response rate and issues with participant availability resulted in a single interview taking place instead. The external service provider was asked similar questions to the case managers but with an emphasis on their perspective working outside the WHSP. The interview transcripts were thematically analysed through open coding. Connections were made across themes, which were then refined to construct the findings.

Quantitative data collection included deidentified administrative data from two sources—Chintaro (social housing data management program) and SHIP (Specialist Homelessness Information Platform). Chintaro records data on housing outcomes such as social housing, private rental, and supported accommodation. SHIP is used by the WHSP to record service user demographic information, presenting issues, support periods and types, and case management data.

Participation in the WHSP is voluntary, so not all women living at the Lakehouse access the program, with 31 out of a possible 40 women choosing to receive support during the evaluation period. Ethics approval was obtained from the RMIT University Human Research Ethics Committee (approved 20/01/2023).

## 4 Constraints

The applicability of the findings from this evaluation are limited, given the small-scale nature of the project. Also, all interviews with service users were conducted while they were residing at the Lakehouse or within a few weeks of leaving. It is therefore not possible to assess longer-term outcomes of the WHSP.

## 5 Key programmatic elements

### 5.1 The sustaining tenancies model

WHSP case management is underpinned by three aims: 1) to settle and stabilise women in the Lakehouse and local community; 2) to access support to secure longer-term affordable and sustainable housing as soon as possible; and 3) to provide settlement support in the new home and community if no other support is available (25). In practical terms, according to a senior manager, this encompasses, ‘supporting [women] to stabilise and then helping them get other long-term housing and really just work through, I guess, next steps for any goals that they're wanting to achieve.’

A program logic map outlining activities, outputs, resources, and outcomes (26) further details these aims. It is premised on the *assumptions for success* that the WHSP model: 1) is evidence-based, trauma-informed, and culturally appropriate; 2) has the necessary expertise, systems, and supports in place to support the model; 3) has established stakeholder and community relationships to facilitate support planning; and 4) ensures community and social supports are responsive, appropriate, and trauma-informed (25). This is achieved through a *sustaining tenancies* model, which is centred on women's identified goals and future plans. The model offers 12-weeks of support, during which time the case managers work with women to accomplish their goals, with a focus on short-, medium-, and longer-term outcomes (see Table 1).

**Table 1:** The WHSP service model (as described by YWCA Australia) (25).

Service user enters the WHSP	Details of program obtained from in-house information session or pamphlet material
First two weeks	Settlement and support plans are developed Agreement on short- and longer-term goals Service users given information on available supports
By six weeks	Settlement requirements are met Support plan goals are progressing Required specialist supports and assistance are accessed Service users feel safe and secure
By twelve weeks	Service users have a record of sustained tenancy at the Lakehouse Long term, stable housing identified Satisfaction with supports received
After leaving the WHSP	Service users have secured long-term, safe affordable housing Service users experience wellbeing and safety in the community Service users connected with and contributing to their community

Since the inception of the WHSP, the sustaining tenancies model has evolved. It was initially delivered as a single period of support over twelve weeks during which support was structured according to formal points in time (as noted in Table 1). At each of these points, women's needs were assessed and it was expected that goals had been achieved. Modifications have now been put in place so that a more flexible approach is applied. For example, multiple periods of support can be offered if required, with periods of support opened and closed according to the individual needs of the woman. As explained by a senior manager:

If a woman actually needs eight weeks of support or needs flexible support […] [it is] closed and open at points when she wants support rather than it being set and you have to engage in support. We did start that at three months and I always said let’s just test it, let’s see what women actually need and want.

Reflective of this needs-responsive model, the evaluation found that the 31 service users received a total of 51 support periods. By offering flexible periods of support, goals can be better tailored to suit service users so that there is no undue pressure placed on achieving outcomes in pre-determined timeframes. Instead, check-ins are used to adapt and respond to changing needs, rather than a set schedule applied to goal achievement measurement. This approach also offers service users greater autonomy in engaging with the WHSP—they can choose when and how they want to participate. One service user, Deborah, described this as, ‘You're already starting on a level playing field [with the workers] where you feel like you want to open up.’

### 5.2 Staffing

At the time of the evaluation, the WHSP was staffed by two part-time case managers (total 7 days per week) and one full-time team coordinator. Case managers work alongside the solo Y housing officer who maintains the Lakehouse tenancy system, deals with maintenance issues, and provides tenancy information and advice to residents. The WHSP team initially experienced disruption in the form of staff turnover and challenges with recruitment. These factors, which occurred during the COVID-19 pandemic, were particularly demanding for a small team trying to manage their caseload in a newly established program. One case manager stated:

When you have a very small team you feel the impact more, especially when you’re setting up a new program or if someone leaves suddenly there’s not the capacity and size of the team to carry that person who’s left, their caseload.

A delay in replacing the team coordinator further compounded the efficiency of the program as this role carries a significant case management load; moreover, it reduced operational oversite. Staff turnover impeded the fostering of strategic relationships with external service providers. These relationships take time to build and are vital in providing older women with pathways to long-term housing and access to support services and community resources. As explained by a senior manager:

It takes time even with staff on board to really develop […] trust and working relationships with housing partners to the point where they want to give rapid housing pathways to the women we’re supporting.

Despite these challenges, senior staff responded swiftly. As one case manager noted, it was ‘quickly resolved within four weeks and we were able to then implement systems—within four weeks of the staffing changes’.

Since the workforce stabilised, the WHSP has been able to implement the model of support thoroughly and to develop strong partnerships with external providers. This was aided by the appointment of skilled staff that have the right experience and knowledge to work successfully with older women experiencing homelessness. It is clear from the evaluation that to be effective in meeting the program goals, the WHSP needs a specialised workforce proficient not only in case management but also in intersectional feminist and trauma-informed practice. This includes understanding and being sensitive to the ways in which women are affected by ageing, domestic/family violence, disability, disease, mental ill health, grief, and trauma. The team demonstrates these capabilities in a manner that is consistently nonjudgmental, flexible and safe, a strength recognised by the participants. Andrea described what this skillset looked like from a service user perspective:

They tread a fine line between formality and informality and they've done that very well. They're happy to sit with you outside so you can have a cigarette while you're talking so I've got nothing but praise for [the case managers], they've done a wonderful job as has everybody here.

### 5.3 Co-location

The WHSP case managers and the Y Housing worker are co-located, dividing their time between the Lakehouse and the YWCA head office. Co-location maximises time and resources by facilitating straightforward referral processes between the two services and coordination of joint activities. This ensures an efficient and smooth experience for service users. A senior manager described this as, ‘It gives them an understanding that we're working together for them as opposed to working in isolation.’ To achieve this, co-location also requires distinct role delineation between the case managers and housing officer, providing clarity on the scope of, and responsibilities attached to, the different roles. As noted by a senior manager:

Residents have an understanding of the roles and responsibilities […] they're not going to the housing officer for the things that they should be going to the Women's Housing Support Program [for].

Support for co-location and role delineation has been formalised through communication protocols between the WHSP and Y Housing: ‘We have to be in communication, so we don't overlap, and we don't step on each other's toes’ (case manager).

## 6 Key supports

### 6.1 Housing

Service users first present to the WHSP seeking assistance with a range of problems. Unsurprisingly, inadequate housing is a key issue. While not unique to older women, this group has distinct support requirements when exiting homelessness. Women in the WHSP described needing housing that accommodated the physical effects of ageing and, for some, the effects of chronic homelessness and rough sleeping, such as disability and/or mobility issues. For those who had experienced domestic/family violence, safety was paramount. Similarly, women experiencing mental ill health require an environment that will not exacerbate their condition. Older women are also more likely to be seeking housing for life, rather than anticipating changing circumstances, such as a growing family, that would necessitate moving to a more suitable dwelling (27, 28). A fundamental service provided by the WHSP is not only finding long-term housing in a severely under-resourced sector, but also sourcing properties that are suitable for older women. A case manager described this as, ‘We're not housing them to just anywhere and everywhere to tick a box […] we're housing them to suit them and to make it last.’ This requires the WHSP to be attentive to individual women's needs and to demonstrate flexibility and patience to ensure the best housing outcomes. Deborah, who was living with mobility issues and had safety concerns related to these, discussed her experience of the WHSP's housing support:

I did find a place but then I heard all bad stories about it. ‘Cause I can't walk and that I didn't want to move there so other than that it just seemed like it's too good to be true, everything, you know what I mean? Yeah. I really appreciate their support and that.

Although the WHSP had found her a newly built property, Deborah did not find the housing suitable and chose not to take it. Despite this, she still felt supported by the WHSP because her case manager was continuing to look for an appropriate property.

### 6.2 Physical health

Support from the WHSP allows women to address physical health needs associated with ageing and that are compounded by, and neglected due to, homelessness. Rebecca was living with several chronic health issues including multiple respiratory and mental health conditions, as well as disabilities that required the use of mobility aids. The WHSP facilitated a referral to a community health centre that addressed Rebecca's health needs that had been long-neglected due to homelessness. This referral resulted in Rebecca receiving support from a physiotherapist, a social worker, and a mental health clinician. She was also supplied with medical aids including a mobility walker and a mobile personal alarm device. Additionally, case managers not only made links with health providers, they also assisted service users such as Rebecca to attend appointments and supported them through their encounters with health professionals.

Rebecca: She came to my first meeting with the social worker and she'd take me into my interviews and stuff, it was brilliant. […] It's like having my best mate because she knew that certain things that upset me and everything else and she'd grab hold of my arm or whatever before I even needed it. But she went out of her way. But it was because we got on so well and what you see is what you get.

Interviewer: Do you think you'd have made it to all those appointments if you hadn't had the case manager taking you?

Rebecca: No, not a chance. No way. No way in the world.

Rebecca described how the trust she built with her case manager was as important as the practical tasks that were undertaken. This relationship was integral to Rebecca being able to follow through with prioritising her health.

### 6.3 Mental health/emotional support

Administrative data indicate that 81 per cent of WHSP servicer users identify as having a mental health condition. After housing, mental health support is the most common reason that service users access the WHSP. Moreover, women who enter the Lakehouse have typically experienced the shock of homelessness for the first time at an older age or have endured the cumulative physical and emotional hardship of chronic homelessness. This is often accompanied by traumatic life events such as family violence, death of a partner, and estrangement from family. Iris had experienced homelessness on and off for many years. She was also a survivor of family violence and had come to the Lakehouse following the death of her partner. Circumstances such as these indicate that access to housing alone may not be enough to provide stability for older women and that mental health and emotional support may also be required. When asked about the type of assistance Iris requested from the WHSP, she responded, ‘Emotional support first of all, I said, and in the long term to find my own place so I can have my own garden.’

The WHSP provides emotional support for service users who may not have other resources available to them. When asked what she had found most useful about being in the WHSP, like Iris, Deborah stated it was the emotional support she received. For Deborah, who had at times experienced suicidal ideation, the quality of this support was demonstrated through the caseworkers’ availability, care, and lack of judgement.

Just knowing that they’re there and they'll listen, you know what I mean? They're not judgemental like just having someone there and I got their number too like knowing that, if you know what I mean, instead of someone's going to judge you or ringing family, you know what I mean. They're just there and they understand your situation as well.

For women such as Iris and Deborah, receiving emotional support is integral to being able to imagine a future with long-term stable housing. Providing emotional support is fundamental to the work undertaken by the WHSP and requires caseworkers who are attuned to the traumatic and gendered life experiences of older women and the challenges they face.

### 6.4 Digital literacy

Accessing long-term housing and seeking support for health and emotional needs can involve navigating complex service systems. This often requires the capability to use digital technologies via mobile phones, computers, and apps. Such systems do not account for people who lack confidence using digital technology, for instance, many older people who do not consider themselves to be digital natives. This can result in older women experiencing homelessness being unable to access essential services. As Deborah explained, she had previously missed medical treatment because she had been unable to log on to online booking systems:

I don’t know how to work the phone; you know that portal for all your appointments and you got to log on and you got to do all this. I don’t know how to do any of that. […] I know I had appointments I missed because I don’t know how to log into this.

Assisting service users with technology, therefore, is a vital service offered by the WHSP. Leah explained how navigating online systems was bewildering without the support of her case worker:

Otherwise I don’t know the system, I don’t know. […] I really can understand and I can feel the support by [my caseworker) working in a system and she put me in another system. It's going from system to system, it’s not me as an outsider coming and jumping into the system.

The administrative burden of digital systems poses a challenge for people in a state of depletion recovering from homelessness. It is a cognitive and emotional demand that they may not have capacity for. Sharon's experience reflects this: ‘I'd still be [in temporary housing] because navigating around paperwork, interviews, everything, emotionally I wouldn't have been able to do it.’ Support with digital technology and systems navigation helps older women to connect with essential services and has a direct impact on their housing and wellbeing outcomes. Without this support from the WHSP, many service users would not have anyone else to assist them and would therefore miss out on critical health and psychological care.

## 7 Discussion

The importance of supporting older women out of homelessness and into permanent, long-term housing using sustained, relational, and holistic practice is demonstrated by the WHSP. The design and service delivery of similar programs can benefit from key elements observed in this model. For example, the program's evolution from a fixed 12-week model to a flexible system of open and closed support periods was a response to the complex and non-linear nature of the lived experience of, and recovery for, older women. Rigid, time-bound support frameworks are unlikely to align with the needs of this cohort, underscored by the data showing 31 service users required 51 distinct periods of support. Rather, flexibility should be built into program logic models so that case managers can pursue service user-identified goals and support women to stabilise in their housing and communities. Case managers were able to integrate this flexible approach into their practice in part due to their specialised expertise. Despite some initial challenges with staff turnover, once settled, the team's specialist knowledge of the intersections of ageing, trauma, mental ill-health, and gender-based violence helped women to feel secure and willing to engage.

Securing housing is by itself not sufficient. The WHSP model shows the importance of an integrated support framework that addresses housing, health, emotional wellbeing, and digital literacy within a context of available and responsive staff teams. Long-term housing stability relies on women being supported to address all these compounding barriers. With the framework of sustaining tenancies, the program prioritised housing that accommodated ageing-related mobility issues and safety concerns, actively supported women to address physical and psychological health needs, accompanied them to appointments to ensure follow-through, recognised the essential role emotional safety played in enabling women to engage in practical tasks, and addressed digital literacy barriers so that women could navigate online medical portals and housing applications. In addition, the strategic advantage of co-location and clear role delineation between housing and support workers had benefits both for the workforce and the women. This intentional design prevented service duplication and overlap, while still facilitating collaboration between the two teams so that referrals and activities could be easily coordinated.

Older women experiencing homelessness are an under-serviced population. Although knowledge of women's homelessness more generally is increasing, the challenges facing older women remain largely unrecognised and specialist services that meet their unique needs are not commonly available. The WHSP community case study offers a much-needed and replicable example of a service that successfully delivers specialist support to older women experiencing homelessness. Through a targeted, flexible, and needs-responsive model of care that is grounded in intersectional feminist and trauma-informed practice, older women experience life-changing housing and support outcomes that would not likely have been available to them via mainstream homelessness services.

## Data Availability

The datasets presented in this article are not readily available because sharing of the dataset has not been approved by the research team. Requests to access the datasets should be directed to juliet.watson@rmit.edu.au.
